# Characteristics of the Donkey’s Dorsal Profile in Relation to Its Functional Body Condition Assessment

**DOI:** 10.3390/ani11113095

**Published:** 2021-10-29

**Authors:** Małgorzata Maśko, Małgorzata Wierzbicka, Łukasz Zdrojkowski, Tomasz Jasiński, Bartosz Pawliński, Małgorzata Domino

**Affiliations:** 1 Department of Animal Breeding, Institute of Animal Science, Warsaw University of Life Sciences (WULS-SGGW), 02-787 Warsaw, Poland; malgorzata_masko@sggw.edu.pl; 2 Department of Large Animal Diseases and Clinic, Institute of Veterinary Medicine, Warsaw University of Life Sciences (WULS-SGGW), 02-787 Warsaw, Poland; malgorzata_wierzbicka@sggw.edu.pl (M.W.); lukasz_zdrojkowski@sggw.edu.pl (Ł.Z.); bartosz_pawlinski@sggw.edu.pl (B.P.)

**Keywords:** equids, posture, shape, landmarks, comparison

## Abstract

**Simple Summary:**

Even though animal posture is known to reflect an emotional state, the presence of chronic postures associated with poor welfare has already been investigated in horses. Measuring, quantifying, and comparing postures requires an application of an objective tool, such as geometric morphometrics. This study aimed to use geometric morphometrics to characterize the dorsal profile of donkeys in relation to their individual features. Forty donkeys were photographed and characterized using the body condition score (BCS), fatty neck score (FNS), dental condition score (DCS), sex, and breed. Then, photographs were analyzed using geometric morphometrics and the differences in dorsal profile between the examined criteria were tested. Within the entire set of donkey dorsal profiles, deformation related to BCS and FNS was observed. FNS measurement seems to have the strongest influence on a donkey’s dorsal profile among the examined criteria. Concluding, the donkeys’ body condition affects their dorsal profile, and both FNS and BCS measurements should be considered when the donkeys’ dorsal profiles are investigated. However, to evaluate the link between the dorsal profile and the welfare state of donkeys, more studies are required.

**Abstract:**

As the breeding of donkeys has increased due to different types of use, welfare evaluation importance increases. This equid’s welfare state has been described using body condition indicators and the geometric morphometrics method. However, the dorsal profile has not yet been assessed in donkeys. In this study, the body condition score (BCS), fatty neck score (FNS), dental condition score (DCS), sex, and breed were used as criteria of dorsal profile deformations. Photographs of 40 donkeys were analyzed using geometric morphometrics. Within the entire set of dorsal profiles, the variance of the first three principal components (PCs) was PC1 = 37.41%, PC2 = 23.43%, and PC3 = 13.34%. The dorsal profiles displayed deformation as an effect of FNS and BCS on size (FNS *p* = 0.012; BCS *p* = 0.024) and shape (FNS *p* < 0.0001; BCS *p* < 0.0001), rather than as an effect of DCS (*p* < 0.0001), sex (*p* = 0.0264), and breed (*p* < 0.0001) only on shape. The highest distances among the categories (Mahalanobis distances: MD ≥ 13.26; Procrustes distances: PD ≥ 0.044) were noted for FNS. The lowest distances were noted between jennets and males (MD = 4.58; PD = 0.012) and between BCS 1 and BCS 2 (MD = 4.70; PD = 0.018). Donkeys’ body condition affects their dorsal profile and both FNS and BCS measurements should be considered when a donkey’s dorsal profile is investigated.

## 1. Introduction

Their set of physiological characteristics and social nature has made donkeys very useful to people in many cultures throughout the ages [1]. These animals nowadays are not an integral part of human life in developed countries; however, currently, a resurgence of interest them can be seen, especially in the dairy industry [2] and in onotherapy [3,4]. As the breeding of donkeys has increased due to different types of use [5], the donkey’s welfare evaluation has become a challenge.

In the welfare evaluation of lactating donkeys, a functional approach to the body condition assessment was introduced [5] as the most practical method to describe the body condition of a donkey. Hitherto, in donkeys, a five-point scale of the body condition score (BCS) was used [6]. Nowadays, the fatty neck score (FNS) enables a complex evaluation of donkeys’ body condition [5]. It has been concluded that FNS, BCS, and the dental condition score (DCS) are necessary to be evaluated simultaneously as an indicator of the donkey’s welfare [5]. As body condition can be considered a key criterion of the overall welfare of the animals [5,6], it became one of the most frequently used morphometric measurements. On the other hand, it has been shown in the study on horses that other morphometric measurements, such as geometric morphometrics (GM), could reflect the posture and hence welfare state of horses [7].

When animal-based morphometric indicators are used to describe an equid’s welfare state, many internal and external features should also be considered. In horses, there are substantial differences in morphometrics between breeds [8], body conditions [9], and mental states [7]. Moreover, the association between the basic animal-based morphometric measurements and the body condition has been evaluated in different studies on ponies, horses [9,10,11], and donkeys [5,12,13]. The association between the animal-based morphometric measurements subjected here has not been evaluated yet, as the knowledge concerning donkeys is still scant. Therefore, we hypothesized that the BCS, FNS, and DCS measurements can impact the GM measurement system used in a previous study on this equid’s posture [14]. Both measurements of the body condition and their potential influence on the donkey’s posture should be considered to understand the complexity of this concept. As the body condition scoring for donkeys [5,6] requires a different technique to that used in horses [9,10,11,15], due to the fat storage in donkeys in more localized areas and a different body shape [6,16], similar species-dependent differences may influence donkey posture.

In the case of posture measurement in horses, the repetition of stressful situations over time may lead to chronic states [17]. Seneque et al. [7] suggested this repetition may lead to the repetition of the same associated postures, which may also become chronic. Authors have been supporting Broom’s theory, in that the effect of the “mental” state of the animal, also reflecting chronic stress, may compound existing health and physical constraints [18]. Seneque et al. showed that the dorsal profile of a horse’s back, such as when it is flat or hollow, is related to a compromised welfare state. Horses with an impaired welfare state and a depression-like syndrome can be differentiated through their overall posture [7]. As the postures and the shape of the dorsal profile of the animals were described as an effective indicator of horses’ [7,14] and pigs’ [19] welfare state, interest in the use of GM in animal breeding has increased. GM is a collection of approaches that provide a mathematical description of the biological forms according to geometric definitions of their size and shape [20]. The GM method uses Landmarks (LD), which are Cartesian coordinates of points in 2D or 3D that can be localized precisely and without ambiguity on a structure and from one specimen to another. For instance, the anatomical points on a body surface reflecting easy-to-find palpation skeleton structures are suitable landmarks in horses [7,14]. Other data points named sliding semilandmarks (SSL) require specific mathematical treatment and are free to slide to capture the geometry of curves and surfaces where landmarks cannot be identified, such as on smooth objects [21]. LD and SSL configurations have been acquired on sets of digital images, and then a Generalized Procrustes Analysis (GPA) is applied to minimize the sum of squared distances between the corresponding LS to extract the shape data by removing the extraneous information of size, location, and orientation [20]. In the general patterns of morphological variation in multidimensional data, conventional Euclidean statistical methods are viable, such as Principal Component Analysis (PCA) [22].

This study aimed to characterize the dorsal profile of donkeys taking into account the typical donkey body condition conformation. To achieve this goal, the relationship between the body condition score, fatty neck score, dental condition score, sex, breed, and the donkey’s general dorsal profile was evaluated using the GM method. Furthermore, some software modifications were introduced to make the GM method more accessible to a growing community of users in various areas of equid breeding and welfare.

## 2. Materials and Methods

### 2.1. Animals

The study involved forty healthy donkeys (n = 40) belonging to five breed groups: Andalusian, Grigio Siciliano, Martina Franca, Magyar Parlagi, and Romanian breeds mixed with the local mix-breed donkeys. The following genotypes could be listed: half-breed Andalusian donkeys (AD, n = 8), half-breed Grigio Siciliano donkeys (GSD, n = 8), half-breed Martina Franca donkeys (MFD, n = 8), half-breed Magyar Parlagi donkeys (MPD, n = 8), and half-breed Romanian donkeys (RD, n = 8). The crosses of breeds were confirmed based on the pedigree data. The donkey group included twenty-one jennets and nineteen males (geldings). In the donkey group, the age (minimum 1.00; 25% percentile 2.00; median 2.00; 75% percentile 4.00; maximum 33.00), height at withers (minimum 104.00; 25% percentile 110.00; median 114.50; 75% percentile 129.80; maximum 138.00), and body weight (minimum 250.00; 25% percentile 292.50; median 300.50; 75% percentile 318.50; maximum 370.00) of the animals were recorded. The donkeys are privately owned and were kept under the same conditions, in the same stable located in southern Poland in Lubachów. All animals were fed three times a day with a personalized dose of meadow hay, water ad libitum, and daily access to grass pastures for no shorter than 8 h per day. Animals did not work and were housed as companion animals. Measurements taken from donkeys were a part of standard veterinary diagnostic procedures, not requiring ethical approval, whereas photographic sessions did not require contact with the animal. During the standard veterinary diagnostic procedures, a basic clinical examination was conducted. The internal temperature, heart rate, respiratory rate, mucous membranes, capillary refill time, and lymph nodes were evaluated following international veterinary standards [23]. A detailed examination of the musculoskeletal system was performed following the guidelines for the lameness evaluation of an athletic horse [24], whereas the mouth examination was performed following the guidelines for a detailed dental examination [25]. Only donkeys showing no clinical signs of any disorders expecting dental disorders were included in the research. None of the donkeys were excluded.

### 2.2. Body Condition Score (BCS) Measures

The BCS was established using a 5-point scale of the previously described scoring system [6]. Four independent researchers (M.M., M.W., Ł.Z., and T.J.) rated the BCS on a scale of 1 (poor) to 5 (obese) after palpation and a visual assessment of the animals. For further analysis, the median of the 4 scores, rounded to the nearest whole or half-score increment, was used. The body weight (BW) was calculated using the previously described formula [26]. For this purpose, body length (OP, olecranon tuber-pin bone) and heart girth (HG) were measured using a soft measuring tape. OP was measured from the olecranon tuber to the ischiatic tuberosity, whereas HG was measured as the circumference of the body, at the point of the olecranon tuber, 2 cm behind the highest point of the withers [5]. The calculated BW was expressed in kg.

### 2.3. Fatty Neck Score (FNS) Measures

**Figure 1:**
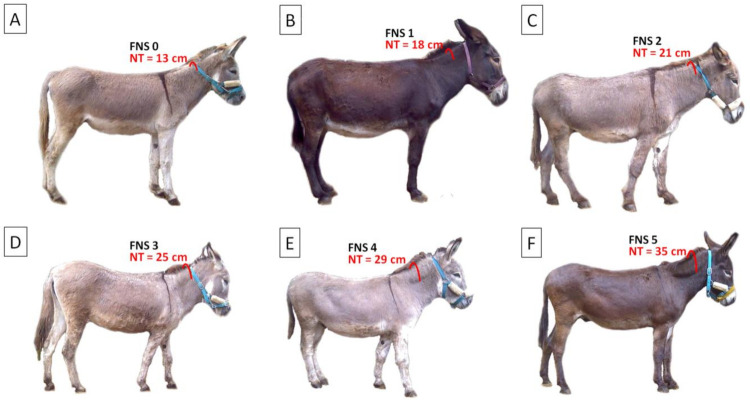
An example of donkeys classified following the fatty neck score (FNS): (**A**) FNS 0, (**B**) FNS 1, (**C**) FNS 2, (**D**) FNS 3, (**E**) FNS 4, and (**F**) FNS 5 categories. The red line indicates measurement of the neck thickness (NT), from one side of the neck to the other at 0.50 of the neck length, taken from the point of the estimated differentiation between the crest and the neck musculature. NT values for individuals are given in cm.

The FNS was determined using the 5-point scale of previously described scoring system [5]. Four independent researchers (M.M., M.W., Ł.Z., and T.J.) rated the FNS on a scale of 1 (poor) to 5 (obese) after palpation and a visual assessment of the animals. For further analysis, the median of the 4 scores, rounded to the nearest whole or half-score increment, was used. The neck thickness (NT) was measured using a soft measuring tape. NT was measured from one side of the neck to the other at 0.50 of the neck length, taken from the point of the estimated differentiation between the crest and the neck musculature [5] (Figure 1).

### 2.4. Dental Condition Score (DCS) Measures

The DCS was determined using a 3-point scale of the previously described scoring system [5]. Four independent researchers (M.M., M.W., Ł.Z., and T.J.) rated the DCS on a scale of 0 (normal dental conditions) to 2 (poor dental conditions) after opening a donkey’s mouth and looking inside for the incisors appearance, palpation of the cheek teeth, and evaluation of an ability to chew. For further analysis, the median of the 4 scores, rounded to the nearest whole or half-score, was used.

### 2.5. Photographs Collection

The GM methodology was adapted from the previously described measuring system of a horse’s posture [7]. Firstly, seven markers on one side of the donkey’s body were positioned. The side of the animal with less mane was selected. The self-adhesive red markers were used to maintain a high contrast with the animal coats. Most of the donkeys had a gray coat on which no gray marker was visible. The markers were used to located anatomical points in the photographs and were positioned from head to hindquarters along the spine, on: the tuber faciale of the corpus maxillae; the articulatio temporomandibularis; the arcus dorsalis of the atlas; the processus transversus of the tenth thoracic vertebra; the articulatio intervertebralis between the last thoracic vertebra and the first lumbar vertebra; the articulatio lumbosacralis; and the first caudal vertebra [14]. The markers were positioned in relation to easy-to-find palpation skeleton structures. The photographs were taken outdoors with no additional lighting.

Afterward, an unfamiliar experimenter led an animal on a slack rope in a walk and stopped gently to achieve spontaneous postures. The animals stopped on flat, hard ground, and were free to stand still and hold their head and neck as they wanted. The experimenter stayed on the left side of the donkey without any voice or body commands, with a slack rope. The photographs were acquired on the left and right sides at a 90° camera angle from a distance of 10 m from the donkey. During each session, photographs of each individual were taken in a standing position (10 photos per individual). The photographs were positioned in the center of the trunk and were taken using a digital camera Canon EOS 5D Mk2 (Canon Inc., Tokyo, Japan) by the same familiar experimenter (MD). As the side of the animal with less mane was selected for photographing, photographs were horizontally turned in order to achieve the same orientation, if required. After the photo was taken, no other corrections such as reduction of noise, sharpness, brightness were utilized. Only photographs on which all four hooves required to be placed completely on the ground were included in the research. The photographs on which a hoof was off the ground or resting as well as on which an animal would not stand still were excluded. Out of a total of 400 photographs, 80 photos (2 photos per individual) were selected for further research based on the inclusion criteria.

### 2.6. Geometric Morphometrics (GM) Measures

**Figure 2:**
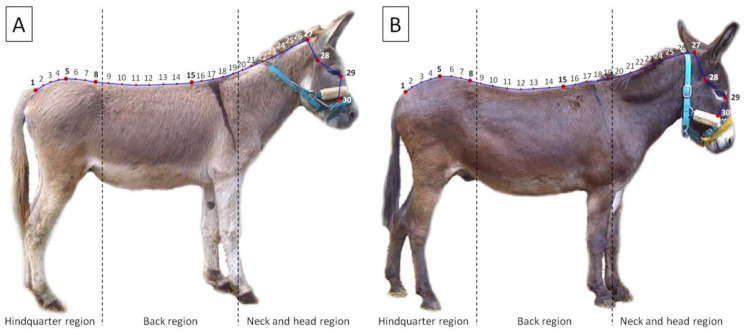
An example of the landmarks (marked with a big red point and bold font) and the sliding semilandmarks (marked with a small red point) digitalization on photography of donkeys classified following the fatty neck score (FNS): (**A**) FNS 0, and (**B**) FNS 5. The blue curves were fitted to the shape of the dorsal profile of animals. In post-processing, the TPS curves were appended to 30 landmarks. Consecutive landmarks are marked with increasing numbers from the first caudalis vertebra (1) to the tuber faciale (30). Dashed lines indicate the boundaries between regions.

Photographs were then analyzed following the previously described protocol [7,14]. The tpsUtil (version 2.31) software was used to build a TPS file containing all the evaluated photo data. The tpsDig2 (version 2.31) software was used to digitized seven landmarks (LD) that reflected the location of the seven markers positioned on a donkeys’ body. The eighth LD was then digitized as the medial canthus of the eye. For the shape analysis, the successive 22 points were digitized using the sliding semilandmarks (SSL) method. The SSL method was used in order to limit as much as possible errors of LD positioning. Four curves with SSL were marked between five consecutive LD, digitized between the arcus dorsalis of the atlas and the first caudal vertebra. Points were added to the curves by length, on the following curves 11 points, 6 points, 2 points, and 3 points, respectively. Using them, the curve was precisely fitted to the shape of the dorsal profile of the animals. The shape obtained in this way kept the anatomical information thanks to the markers and then the LD. All LD and SSL were digitized by the same experimenter (TJ) following the same order to avoid a spurious superimposition during the Procrustes fit. Since all photos were taken using the same settings and under the same condition, the same scale factor to all of them was applied. The tpsUtil (version 2.31) software was then used to append the TPS curve to the landmarks, which allowed to obtain 30 LD reflecting the shape of the dorsal profile. Some LD were grouped to focus just on the hindquarter (LD 1 to 8, from the first caudalis vertebra to the articulatio intervertebralis between the last thoracic vertebra and the first lumbar vertebra), back (LD 9 to 19, from the above articulatio intervertebralis to the fourth LD caudally from the tenth thoracic vertebra), or neck and head (LD 20 to 30, from the above fourth LD to the tuber faciale of the corpus maxillae) (Figure 2).

The single TPS file received contained ID information about the sex, breed, BCS, FNS, and DCS. MorphoJ software (Copyright 2008–2019 Christian Peter Klingenberg, Licensed under the Apache License, Version 2.0, https://morphometrics.uk/MorphoJ_guide/frameset.htm?index.htm, accessed on 28 October 2021), an integrated software package for geometric morphometrics, was used for further analyses [27]. MorphoJ (version 2.0) is preferred to R packages since it is probably the easiest standalone software to use [20]. First, the extraction of a new classifier from ID strings was performed to classify the sex, breed, BCS, FNS, and DCS. The Generalized Procrustes Analysis (GPA, returning Procrutes coordinates), Covariance matrix (CovMatrix) generation, and Principal Component Analysis (PCA) were then conducted to visualize the distribution of the shape configurations corresponding to the donkey’s postures. The classifier variables were used to determine the color for each category on a scatter plot of the principal component scores. The confidence ellipses were drawn using a 0.9 probability and a classifier as a criterion for grouping the observations. The classifier was also used to determine the colors of the ellipses and data points.

### 2.7. Data Analysis

The shape of the dorsal profile of the donkeys was studied on the first three principal components (PC) resulting from the PCA. The principal component scores were then grouped and colored for each category determined based on the classifier variables: sex, breed, BCS, FNS, and DCS. Average observations for each category were executed and displayed as wireframe graphs and transformation grids. The Procrustes ANOVA was used to determine the classifiers’ (sex, breed, BCS, FNS, and DCS) effect on the centroid size and shape, with the significance level established as *p* < 0.05. The Canonical Variate Analysis (CVA) and the Angular Comparison of Vector Directions (ACVD) method were then applied to the determined distances (Mahalanobis distances, MD; Procrustes distances, PD) for dorsal profiles among the examined categories, respectively. All analyses and visualizations were performed using MorphoJ version 2.0 software.

## 3. Results

The entire dorsal profile of the donkeys was first investigated to try to identify variations in postures associated with the previously reported body condition indicators. The characteristic postures for sex, breed, BCS, FNS, and DCS classes and the direction of changes in the hindquarter, back, and neck and head regions were then examined.

**Figure 3:**
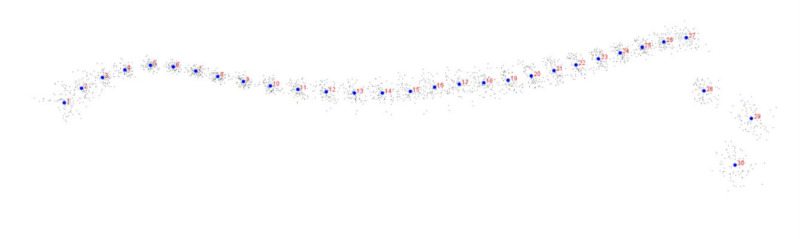
Procrustes coordinates of the donkey data set. Blue points represent the consecutive landmarks marked with increasing numbers from the first caudalis vertebra (1) to the tuber faciale (30).

**Figure 4:**
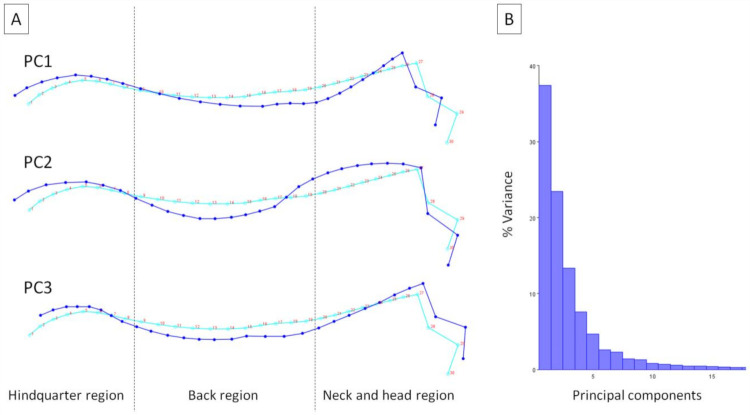
Principal components of the dorsal profiles of donkeys, represented by (**A**) the wireframe graph and (**B**) the histogram of variance. Light blue landmarks and curves represent the consensus donkey’s dorsal profile. Dark blue landmarks and curves represent the extremum (minimum of the axis) of PC1, PC2, and PC3, respectively. Dashed lines indicate the boundaries between regions. Consecutive landmarks are marked with increasing numbers from the first caudalis vertebra (1) to the tuber faciale (30).

Procrustes coordinates for all individuals are presented in Figure 3. PCs represent the weight of the partial wraps in the whole warps between all the conformations. The extremum of PC1 supported the dorsal profile with an elevated dorsal line in the hindquarter region, lowered dorsal line of wither in the back region, and elevated occiput in the neck and head region in relation to the consensus dorsal profile. The extremum of PC2 supported the dorsal profile with an elevated dorsal line in the hindquarter region, lowered dorsal line in the back region, and elevated dorsal line in the neck and head region in relation to the consensus dorsal profile. The extremum of PC3 supported the dorsal profile with an elevated and shortened dorsal line in the hindquarter region, lowered dorsal line in the back region, as well as stretched forward and elevated occiput in the neck and head region in relation to the consensus dorsal profile (Figure 4A). The variance of the first three PCs was as follows: PC1 = 37.41%; PC2 = 23.43%; and PC3 = 13.34% (Figure 4B).

**Figure 5:**
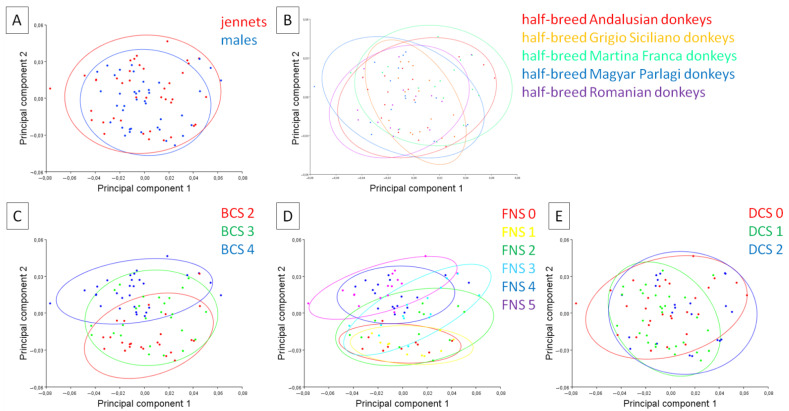
A scatter plot of the principal component scores of the donkeys. The color for each category was determined based on the classifier variables: (**A**) sex (jennets; males); (**B**) breed (half-breed Andalusian donkeys, half-breed Grigio Siciliano donkeys, half-breed Martina Franca donkeys, half-breed Magyar Parlagi donkeys, and half-breed Romanian donkeys); (**C**) body condition score (BCS 2, BCS 3, and BCS 4); (**D**) fatty neck score (FNS 0, FNS 1, FNS 2, FNS 3, FNS 4, and FNS 5); and (**E**) dental condition score (DCS 0, DCS 1, and DCS 2). The confidence ellipses were drawn using a 0.9 probability and a classifier as a criterion for grouping the observations.

Five classifiers were used to distinguish the categories of individuals, including sex (21 jennets and 19 males), breed (8 half-breed Andalusian donkeys, 8 half-breed Grigio Siciliano donkeys, 8 half-breed Martina Franca donkeys, 8 half-breed Magyar Parlagi donkeys, and 8 half-breed Romanian donkeys), BCS (12 donkeys with BCS 2, 13 with BCS 3, and 15 with BCS 4, no donkey was rated poor (BCS 1) or obese (BCS 5)), FNS (5 donkeys with FNS 0, 5 donkeys with FNS 1, 6 donkeys with FNS 2, 7 donkeys with FNS 3, 9 donkeys with FNS 4, and 8 donkeys with FNS 5), and DCS (14 donkeys with DCS 0, 15 donkeys with DCS 1, and 11 donkeys with DCS 2). On scatter plots of principal component scores in the PC1 to PC2 orientation, it is easy to see that the scores are not divided into separate categories of sex (Figure 5A), breed (Figure 5B), and DCS (Figure 5E). On the other hand, the scores are partially divided into separate categories of BCS (Figure 5C) and FNS (Figure 5D). More donkeys with BCS 2 and FNS 0–1 represented the dorsal profile supported by PC1, whereas more donkeys with BCS 4 and FNS 4 and 5 represented the dorsal profile supported by PC2.

The Procrustes ANOVA was applied to assess the effect of the examined classifiers on both the centroid size and shape of the donkey’s dorsal profile. The donkey’s dorsal profile showed significant differences for size depending only on the BCS (*p* = 0.024) and FNS (*p* = 0.012), whereas for shape, depending on all classifiers: sex (*p* = 0.0264), breed (*p* < 0.0001), BCS (*p* < 0.0001), FNS (*p* < 0.0001), and DCS (*p* < 0.0001) (Table 1).

**Figure 6:**
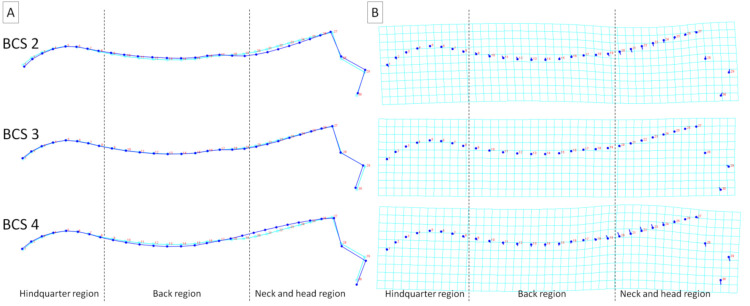
Average observations of the donkeys’ dorsal profiles grouped by body condition score (BCS 2, BCS 3, and BCS 4) and represented by (**A**) wireframe graphs and (**B**) a transformation grid. In the wireframe graphs, light blue landmarks and curves represent the consensus donkey’s dorsal profile and the dark blue landmarks and curves represent the average observations for the subsequent BCS scores. On the transformation grid, dark blue landmarks represent the consensus donkey’s dorsal profile as well as dark blue lines represent the average observations for subsequent BCS scores. Dashed lines indicate the boundaries between regions. Consecutive landmarks are marked with increasing numbers from the first caudalis vertebra (1) to the tuber faciale (30).

**Figure 7:**
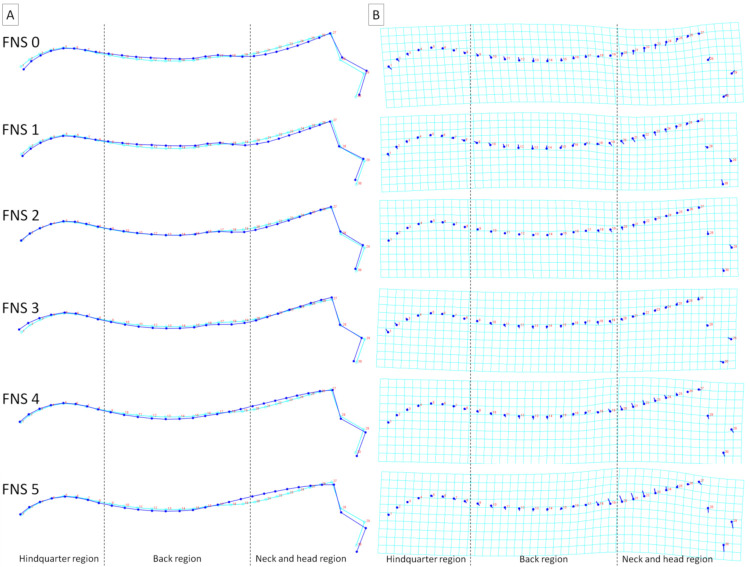
Average observations of the donkeys’ dorsal profiles grouped by fatty neck score (FNS 0, FNS 1, FNS 2, FNS 3, FNS 4, and FNS 5) and represented by (**A**) wireframe graphs and (**B**) a transformation grid. In the wireframe graphs, light blue landmarks and curves represent the consensus donkey’s dorsal profile and the dark blue landmarks and curves represent the average observations for the subsequent FNS scores. On the transformation grid, dark blue landmarks represent the consensus donkey’s dorsal profile and the dark blue lines represent the average observations for the subsequent FNS scores. Dashed lines indicate the boundaries between regions. Consecutive landmarks are marked with increasing numbers from the first caudalis vertebra (1) to the tuber faciale (30).

**Figure 8:**
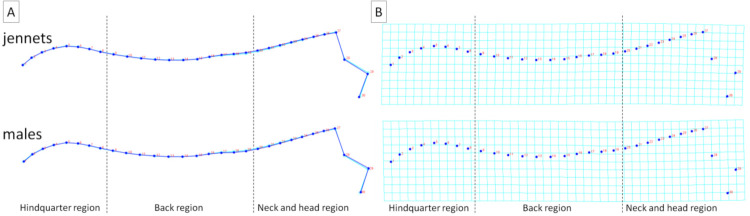
Average observations of the donkeys’ dorsal profiles grouped by sex (jennets; males) and represented by (**A**) wireframe graphs and (**B**) a transformation grid. In the wireframe graphs, light blue landmarks and curves represent the consensus donkey’s dorsal profile and the dark blue landmarks and curves represent the average observations for the subsequent sex groups. On the transformation grid, dark blue landmarks represent the consensus donkey’s dorsal profile and the dark blue lines represent the average observations for the subsequent sex groups. Dashed lines indicate the boundaries between regions. Consecutive landmarks are marked with increasing numbers from the first caudalis vertebra (1) to the tuber faciale (30).

**Figure 9:**
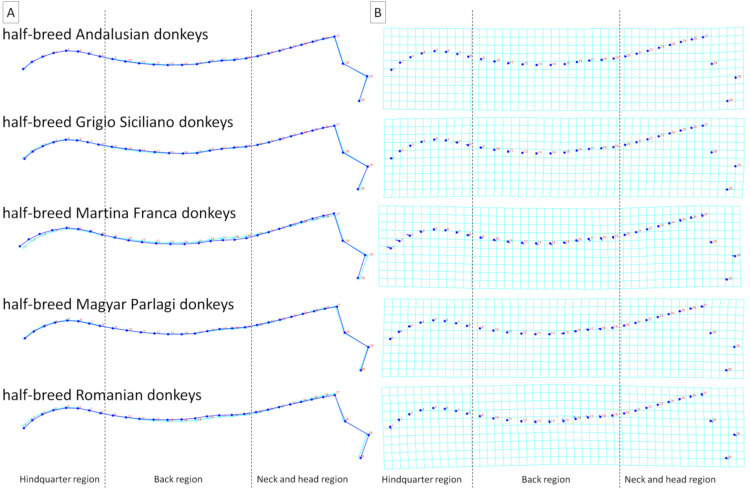
Average observations of the donkeys’ dorsal profiles grouped by breed (half-breed Andalusian donkeys, half-breed Grigio Siciliano donkeys, half-breed Martina Franca donkeys, half-breed Magyar Parlagi donkeys, and half-breed Romanian donkeys) and represented by (**A**) wireframe graphs and (**B**) a transformation grid. In the wireframe graphs, light blue landmarks and curves represent the consensus donkey’s dorsal profile and the dark blue landmarks and curves represent the average observations for the subsequent breed groups. On the transformation grid, dark blue landmarks represent the consensus donkey’s dorsal profile and the dark blue lines represent the average observations for the subsequent breed groups. Dashed lines indicate the boundaries between the regions. Consecutive landmarks are marked with increasing numbers from the first caudalis vertebra (1) to the tuber faciale (30).

**Figure 10:**
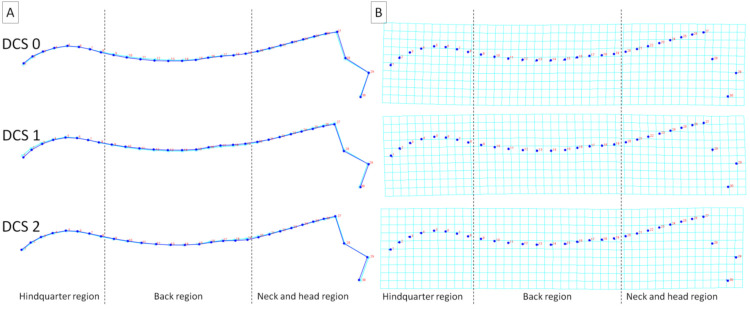
Average observations of the donkeys’ dorsal profiles grouped by body dental condition score (DCS 0, DCS 1, and DCS 2) and represented by (**A**) wireframe graphs and (**B**) a transformation grid. In the wireframe graphs, light blue landmarks and curves represent the consensus donkey’s dorsal profile and the dark blue landmarks and curves represent the average observations for the subsequent DCS scores. On the transformation grid, dark blue landmarks represent the consensus donkey’s dorsal profile and the dark blue lines represent the average observations for the subsequent DCS scores. Dashed lines indicate the boundaries between regions. Consecutive landmarks are marked with increasing numbers from the first caudalis vertebra (1) to the tuber faciale (30).

To better visualize the differences in dorsal profiles between the examined categories of individuals, average observations for consecutive classifiers were executed. These visualizations confirm the results obtained for principal component scores, where the features of the division into separate categories were higher for BSC and FNS (Figure 6 and Figure 7) than for sex, breed, and DCS (Figure 8, Figure 9 and Figure 10). It is easy to see that for BCS 2, FNS 0, and FNS 1, a dorsal profile with an elevated dorsal line of the wither in the back region and lowered dorsal line in the neck and head region was observed. On the other hand, for BCS 4, FNS 4, and FNS 5, a dorsal profile with an elevated dorsal line in the neck and head region was noted. No other pronounced deformations for BCS 3, FNS 2, and FNS 3, nor for the sex groups, breed groups, and DCS scores were observed.

As a summary of the recent results, the distances between the donkeys’ dorsal profiles were compared among the BCS (Table 2), FNS (Table 3), sex (Table 4), breed (Table 5), and DCS (Table 6) categories. The highest distances among the categories (Mahalanobis distances ≥ 13.26 and Procrustes distances ≥ 0.044) were noted for FNS between FNS 1 and FNS 5, FNS 0 and FNS 5, as well as FNS 1 and FNS 4. The lowest distances among the categories (Mahalanobis distances ≤ 5.24 and Procrustes distances ≤ 0.018) were noted for sex between jennets and males, for BCS between BCS 1 and BCS 2, as well as for breed between half-breed Martina Franca donkeys and half-breed Romanian donkeys.

## 4. Discussion

An analysis of the dorsal profiles of the examined donkeys displayed a deformation associated with the body condition indicators, this being higher in the functional one (FNS; effect on size *p* = 0.012; effect on shape *p* < 0.0001) [5] rather than the most frequently used one (BCS; effect on size *p* = 0.024; effect on shape *p* < 0.0001) [6,9,10,11,12]. In donkeys, BCS is proposed as an index of the overall adiposity [6,12], whereas FNS is a morphometric index of regional fat deposition [5]. Since the adipose tissue of donkeys tends to cumulate in the neck region and droop on both sides of the crest of the neck [6], it is not surprising that FNS had a stronger influence on the dorsal profile than BCS. Since in this study no donkey was rated poor (BCS 1) or obese (BCS 5), the effect of BCS may be stronger when the full range of the scale will be used. However, in donkeys, this regional adiposity could play a different role than the indicator of the metabolic status, which is used in horses and ponies [9,28], and it can remain even when the overall body weight decreases [6,16]. Therefore, the results of the present study supported Valle et al.’s findings that FNS is important for the description of the body conformation of donkeys [5].

It should be kept in mind that donkeys are not small horses, although they both belong to the Equidae family [16]. The shape of the neck and back of a donkey is different from that of a horse. The donkey’s general vertebral formula is C7, T18, L5, S5, Cd15–17 [29], whereas in the horse it is C7, T18, L6, S5, Cd15–21 [30]. The shorter lumbar vertebrae and relatively shorter neck in the donkey than in the horse support a heavy skull [16]. Therefore, in donkeys, a remarkably thick cutaneus colli muscle covers the middle one-third of the length of the neck and is evenly developed [31]. Moreover, donkeys are at greater risk of obesity compared to horses [6]. Therefore, the enlarged and thickened neck with the longitudinal fat deposits in the crest located on both sides of the neck [5] may deform the dorsal profile similar to a “depressed” or “abnormal” posture in horses [7]. In a recent study on horses, a flat or hollow dorsal profile was related to a compromised welfare state [7], whereas in this study a similar posture was associated with a low body condition. As body condition can be considered a key criterion of the overall welfare of the animals [5], application of a posture analysis as an additional indicator of equids’ welfare [7] should in donkeys show the impact of regional fat storage on the neck region, which is reported here.

In the present study, no significant evidence of an association between deformations in the size of the dorsal profile and sex (*p* = 0.100), breed (*p* = 0.614), or DCS (*p* = 0.554) was noted. On the other hand, evidence of an effect of sex (*p* = 0.026), breed (*p* < 0.0001), and DCS (*p* < 0.0001) on the shape of the dorsal profile were observed. These findings are partially in contradiction and partially in agreement to the Valle et al. study [5], where dental disorders were considered an indicator of the body condition for lactating donkeys. Other recent studies have demonstrated the association between DCS and BCS or weight loss [32,33]. However, based on the results of the present study, no significant association can be established between DCS and deformation in the size of the dorsal profile (*p* = 0.554), whereas a significant association can be established between DCS and deformation of the shape of the dorsal profile (*p* < 0.0001). Results of the present study are also partially in contradiction and partially in agreement to Mendoza et al.’s study [34], where jennets had significantly greater body measurements, including BCS, than males, and showed a tendency to have higher triglyceride concentrations. Although the relationship between DCS and BCS as well as between sex and BCS was not determined in this study, the observed lack of deformation in the size of the dorsal profile (*p* = 0.100) may be due to the limited size of the groups, which should be considered as one of the limitations of this study. The second limitation of this study is the use of non-purebred donkeys, but a crossbreed of breeds. Therefore, the intraspecific variability in anatomy and physical conformations, which is typical for donkeys [16,35], did not appear in this study as a classifier for deforming the dorsal profile. In this study, it is important that the evaluation of the group of donkeys was kept under the same conditions—in the same stable and managed in the same way. Therefore, it was decided to use all available donkeys, which were not representing pure breeds. However, one should not forget that between the donkey breeds, huge interbreed morphotype variability occurred [35,36]. Andalusian donkeys are disease and heat resistant and full of energy, with a calm and balanced temperament. These are large-sized donkeys, on average 146–155 cm in height at withers [35]. They have an impressive head, with roman noses, very large ears, and a muscular neck [36]. Martina Franca donkeys are relatively large-sized donkeys, on average 127–135 cm in height at withers. They have a large head with a well-developed, strong, and muscular neck, and a large, long, and muscular croup [35]. Magyar Parlagi donkeys is a Hungarian Steppe Donkey that come in two varieties; a small size, on average 110–115 cm in height at withers, and a large size, on average 136–145 cm in height at withers [36]. Romanian donkeys are characterized by a large variation in body length with no standardized breed height. Yılmaz et al. [36] suggested the reason of such variability may be due to the change in confirmation from youth to maturity. In turn, the height of the Grigio Siciliano donkeys is undescribed [36].

For large population studies and the increasing availability of research methods, some software modifications were introduced here. The labels were digitized following the SSL method described by Seneque et al. [14]; however, MorphoJ software was used for all analyses [27], rather than other reported morphometric-based software, such as R Core Team (2018) packages (‘geomorph,’ ‘shapes,’ ‘Morpho,’ and ‘Momocs’) [20]. MorphoJ software was used as probably the easiest standalone software for the GM method. The graphical user interface is simple and clear, and one can quickly run several analyses and generate fully customized graphs that can be exported as images or vectorized figures [20]. The only insufficient aspect is a display of one extremum of the PCs rather than two and the inability to cancel the movement of the neck. In recent studies, the movement of the neck, corresponding to a rotation around the withers, was canceled thanks to the R library geomorph [7,14]. In this study, the movement of the neck corresponding to a rotation in the articulatio atlantooccipitalis was not canceled; however, no significant deformation of the dorsal profile was found in this region. Since all body condition-related deformations were described for the neck regions, the potential rotation in the atlas area should not affect future research; therefore, the correction of head position, which is recommended in scientific studies, may be denned into this more practical one. This inconvenience is disproportionately small compared to the benefits of facilitating statistical analyses in MorphoJ software. We hope this modification will increase the availability of the GM method to a growing community of users in various areas of management of breeding and welfare evaluation of donkeys.

In summary, it can be stated that the donkey’s dorsal profile allows distinguishing categories of individuals, used so far in the welfare evaluation of lactating donkeys [5], and thus could be a promising indicator of welfare states, similarly to horses [7]. Nevertheless, further studies are needed to investigate whether there is a link between the deformation of the dorsal profile and the poor welfare state of donkeys.

## 5. Conclusions

A characteristic of the dorsal profile of donkeys has been proposed in the present study. The results underline the fact that donkeys’ body condition affect their dorsal profile. In donkeys, the functional approach to body condition measurement (FNS) rather than the one most frequently used (BCS) should be considered when the dorsal profile is investigated. However, in order to evaluate the link between the deformation of the dorsal profile and the poor welfare state of donkeys, more studies are required. We hope the simplification of the geometric morphometrics software, described in this paper, will popularize the dorsal profile evaluation in various areas of management of donkeys’ breeding and welfare evaluation. We also hope the results of this study may improve further research on a donkeys’ welfare state.

## Figures and Tables

**Table 1:** The effect of classifiers (sex, breed, BCS, FNS, and DCS) on both the centroid size and shape of a donkey’s dorsal profile, determined using the Procrustes ANOVA by sums of squares (SS) and mean squares (MS). The significance level was established as *p* < 0.05. The significant effect of the classifier is marked in bold font in the *p*-value column. The bold is used here to separate the heading and the features/data.

Centroid Size	SS	MS	df	F	*p*
sex	140,716.4	140,716.4	1	2.77	0.1
breed	141,668.6	35,417.14	4	0.67	0.614
BCS	380,640.3	190,320.1	2	3.94	0.024
FNS	275,181.6	55,037.12	5	1.06	**0.012**
DCS	62,500.1	31,250.05	2	0.6	**0.554**
**Shape**	**SS**	**MS**	**df**	**F**	***p***
sex	0.003	0.00004	56	1.4	**0.026**
breed	0.012	0.00005	224	1.6	**<0.0001**
BCS	0.023	0.0002	112	6.79	**<0.0001**
FNS	0.039	0.0001	280	4.99	**<0.0001**
DCS	0.008	0.00007	112	2.05	**<0.0001**

**Table 2:** Mahalanobis distances (MD) and Procrustes distances (PD) among the body condition score (BCS) categories (BCS 1, BCS 2, and BCS 3).

		BCS 1	BCS 2
BCS 2	MD	4.7	
	PD	0.018	
BCS 3	MD	7.13	5.23
	PD	0.039	0.027

**Table 3:** Mahalanobis distances (MD) and Procrustes distances (PD) among the fatty neck score (FNS) categories (FNS 0, FNS 1, FNS 2, FNS 3, FNS 4, and FNS 5).

		FNS 0	FNS 1	FNS 2	FNS 3	FNS 4
FNS 1	MD	7.01				
	PD	0.018				
FNS 2	MD	7.38	7.41			
	PD	0.022	0.019			
FNS 3	MD	8.07	9.02	5.37		
	PD	0.037	0.032	0.017		
FNS 4	MD	11.75	13.26	9.91	8.92	
	PD	0.041	0.044	0.032	0.026	
FNS 5	MD	13.99	16.37	12.83	10.89	5.54
	PD	0.051	0.053	0.043	0.038	0.015

**Table 4:** Mahalanobis distances (MD) and Procrustes distances (PD) among the sex categories (jennets and males).

		Jennets
Males	MD	4.58
	PD	0.012

**Table 5:** Mahalanobis distances (MD) and Procrustes distances (PD) among the breed categories (half-breed Andalusian donkeys, AD; half-breed Grigio Siciliano donkeys, GSD; half-breed Martina Franca donkeys, MFD; half-breed Magyar Parlagi donkeys, MPD; and half-breed Romanian donkeys, RD).

		AD	GSD	MFD	MPD
GSD	MD	5.72			
	PD	0.01			
MFD	MD	6.36	5.6		
	PD	0.019	0.023		
MPD	MD	6.65	6.33	7.03	
	PD	0.013	0.015	0.028	
RD	MD	5.37	5.84	5.24	6.24
	PD	0.03	0.017	0.013	0.018

**Table 6:** Mahalanobis distances (MD) and Procrustes distances (PD) among the dental condition score (DCS) categories (DCS 0, DCS 1, and DCS 2).

		**DCS 0**	**DCS 1**
DCS 1	MD	5.36	
	PD	0.016	
DCS 2	MD	6.4	5.56
	PD	0.019	0.017

## Data Availability

The data presented in this study are available on request from the corresponding author.
